# The Spectrum of Risk Factors for Falls is Broader Than Expected

**DOI:** 10.1002/hsr2.70582

**Published:** 2025-03-18

**Authors:** Josef Finsterer

**Affiliations:** ^1^ Neurology Department Neurology & Neurophysiology Center Vienna Austria

## Conflicts of Interest

The author declares no conflicts of interest.

Dear Editor,

We were interested to read the article by Rahimi et al. about a study on the risk factors for falls in rehabilitation patients and the development and validation of the Fall Risk Assessment Scale (FRAS) [1]. Falls were found to be related to comorbidities, current medication, fall history, cognition, and three items of the Functional Independency Measure (FIM) [1]. Patients with a history of falls had a higher risk of falling than non‐fallers [1]. The study is excellent, but some points should be discussed.

The first problem is that several risk factors for falls were not included in the assessment [1]. These include fluid intake, blood pressure, heart failure, bradycardia, atherosclerosis, carotid stenosis, endocrine disorders (e.g., hypoaldosteronism, diabetes), metabolic disorders (e.g., acidosis, alkalosis) or renal disorders (e.g., hypokalaemia, polyurea). To assess which risk factors for falls were really included after the first study phase, it would be helpful to know the detailed list of risk factors extracted for the Fall Risk Assessment Scale (FRAS). Although “neurological disorders” were included in the assessment of fall risk factors, it was not reported whether small or large fiber neuropathies were included in the risk assessment. Since patients with sensory disturbances are predisposed to dizziness and thus to falls, sensory disturbances at all levels must be included in the analysis.

The second problem is that not only anti‐seizure drugs, tranquilizers, or antihypertensives are at risk for falls, but also a number of other drugs such as neuroleptics, anxiolytics, hypnotics, diuretics, antiarrhythmics, antihistamines, antirheumatics, and some anticancer drugs. The entire spectrum of medications that impair vision, concentration, alertness and orientation should also be known and must be ruled out as a cause of falls. Alcoholism and illegal drugs must also be ruled out as a cause of falls.

The third point is that it is not only patients in rehabilitation facilities who fall frequently, but also patients who are admitted to hospital, depending on the underlying disease and hospital ward. Parkinson's patients and patients with cardiomyopathy or neuromuscular diseases and muscle weakness also frequently fall at home.

The fourth point is that it is incomprehensible why stroke, spinal cord injuries, and brain injuries should not be counted as neurological diseases, as mentioned in the introduction [1]. This should be corrected.

The fifth point is the retrospective design of phase 1 of the study. Retrospective designs have several limitations. Some data may be missing, the accuracy of the data cannot be easily verified, desired missing or new data can no longer be generated, references to specific studies are often untraceable, and follow‐up studies are not feasible.

In summary, this interesting study has limitations that put the results and their interpretation into perspective. Addressing these limitations could strengthen the conclusions and support the message of the study. All unresolved issues need to be clarified before readers can uncritically accept the study's message. When assessing the risk factors for falls and validating the FRAS, all influencing factors must be included in the analysis.

## Author Contributions

**Josef Finsterer:** conceptualization, data curation, formal analysis, resources, writing – original draft, writing – review and editing.

## Ethics Statement

The author has nothing to report.

## Consent

The author has nothing to report.

## Data Availability

All data are available from the corresponding author.
